# Dual Antiviral and Immunomodulatory Effects of Phytochemicals in Influenza A Virus Infection: Targeting Key Host Signaling Pathways

**DOI:** 10.1002/mbo3.70424

**Published:** 2026-09-28

**Authors:** Dibakar Chowdhury, Hillary A. Vanderven, Phurpa Wangchuk, Subir Sarker

**Affiliations:** ^1^ Biomedical Sciences & Molecular Biology, College of Medicine and Dentistry James Cook University Townsville Queensland Australia; ^2^ Australian Institute of Tropical Health and Medicine James Cook University Townsville Queensland Australia; ^3^ Department of Microbiology and Immunology, Peter Doherty Institute for Infection and Immunity The University of Melbourne Parkville Victoria Australia; ^4^ College of Science and Engineering James Cook University Cairns Queensland Australia; ^5^ Australian Institute of Tropical Health and Medicine James Cook University Cairns Queensland Australia; ^6^ Department of Microbiology, Anatomy, Physiology and Pharmacology, School of Agriculture, Biomedicine and Environment La Trobe University Melbourne Victoria Australia

**Keywords:** influenza A virus, innate immunity, MAPK, NF‐κB, phytochemicals, RIG‐I, TLR

## Abstract

Influenza A virus (IAV) remains a major public health threat due to its high genetic variability and ability to evade host immune responses. While antiviral drugs and vaccines remain the key measures against influenza infection, their limited efficacy and the emergence of resistant viral strains have prompted growing interest in exploring complementary therapeutic approaches. In this review, we critically evaluate current evidence on how plant‐derived compounds modulate innate immune signaling networks, including toll‐like receptor, retinoic acid‐inducible gene I, mitogen‐activated protein kinase, and nuclear factor kappa B pathways during IAV infection. We further address the pathway cross‐talk that underpins this broad immunomodulatory activity, the translational barriers including bioavailability, metabolic transformation, and standardization that currently limit clinical progression, and the mechanistic gaps that future studies must resolve. Collectively, this review argues that phytocompounds should be understood as modulators of an integrated innate immune network, an insight that reframes both their therapeutic potential and the experimental standards required to advance them.

## Graphical abstract

Influenza A virus modulates host innate immune pathways, including TLR, RIG‐I, MAPK, and NF‐κB signaling, to support replication and drive inflammatory pathology. Plant‐derived phytocompounds target these interconnected pathways, offering a potential host‐directed immunomodulatory approach complementary to conventional antivirals. However, their clinical translation remains limited by incompletely defined molecular targets, poor bioavailability, unresolved safety profiles, and lack of pharmaceutical standardization.

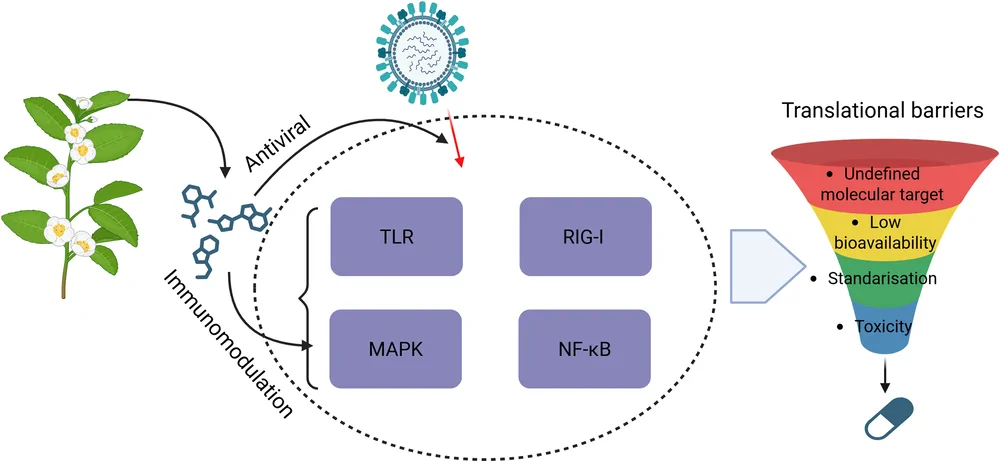

## 1 Introduction

Influenza is an acute respiratory infection caused by influenza viruses, which are enveloped, negative‐sense RNA viruses belonging to the family *Orthomyxoviridae* (Palese 2004). Owing to their remarkable genetic plasticity, driven by antigenic drift and antigenic shift, these viruses continually generate novel variants capable of evading pre‐existing immunity, posing an ongoing global health challenge (Webster et al. 1992). Seasonal influenza A virus (IAV) subtypes, such as H1N1 and H3N2, remain the predominant circulating strains, collectively infecting up to one billion people and causing an estimated 290,000–650,000 deaths annually worldwide (WHO 2025).

Four antiviral drugs are approved by the U.S. Food and Drug Administration (FDA) for influenza treatment, including oseltamivir, zanamivir, peramivir, and baloxavir marboxil. Adamantanes (amantadine and rimantadine), once used as M2 ion channel blockers, are no longer recommended due to widespread resistance among circulating IAVs (Li et al. 2023). Although oseltamivir, the first orally available neuraminidase (NA) inhibitor, remains a widely prescribed drug, the emergence of resistance to NA inhibitors, including oseltamivir and zanamivir, continues to raise concern about therapeutic efficacy (Thorlund et al. 2011). Nevertheless, the number of distinct small‐molecule antiviral classes widely approved for influenza remains limited, underscoring a major gap in therapeutic options (Hayden and Shindo 2019).

For centuries, bioactive compounds derived from fresh plant materials and solvent‐based extracts have played an integral role in traditional and modern medicines across diverse cultures. These natural products represent a vast and largely untapped source of structurally diverse molecules with potential pharmacological activity (Mohanty et al. 2023). Oseltamivir, a widely used antiviral, is a semi‐synthetic derivative of shikimic acid, which is naturally obtained from the plant *Illicium verum* (star anise). This illustrates the important contribution of plant‐derived compounds to the development of modern antiviral drugs (El Sayed 2000; Kim and Park 2012). Therefore, in recent years, increasing attention has been directed toward plant‐derived compounds with immunomodulatory properties that not only interfere with viral replication but also regulate host immune responses (Arumugam et al. 2025). This approach is particularly relevant for influenza virus infection, which can be characterized by immune dysregulation, notably excessive cytokine production, or “cytokine storm,” that contributes more to pulmonary pathology and mortality than direct viral damage (Chon 2012). Central to these processes are key signaling cascades, including Toll‐like receptors (TLRs), retinoic acid‐inducible gene I (RIG‐I) like receptors, mitogen‐activated protein kinase (MAPK) pathways, and nuclear factor kappa B (NF‐κB), which orchestrate antiviral responses through interferon (IFN) and cytokine induction, but may also drive immunopathology when aberrantly activated (Pizzolla et al. 2017).

A growing body of research studies indicates that plant bioactive compounds, such as flavonoids, alkaloids, terpenoids, polysaccharides, and phenolic acids, can modulate these immune pathways in both in vitro and in vivo models. For example, several flavonoids suppress TLR3‐ or TLR7‐mediated signaling, inhibit NF‐κB and MAPK hyperactivation, and fine‐tune IFN responses, thereby reducing excessive pro‐inflammatory cytokine production while preserving antiviral defense (Arumugam et al. 2025). This dual functionality, direct antiviral activity coupled with host immunomodulation, positions plant‐derived compounds as promising candidates for adjunctive influenza therapy. Nevertheless, significant gaps remain: many studies focus primarily on immune modulation without assessing viral replication dynamics, while others employ crude extracts where active components and mechanisms of action are insufficiently characterized (Sen et al. 2021; Lu et al. 2025). Furthermore, the translation of these preclinical findings into clinical efficacy remains limited.

In this review, we summarize current research findings on plant‐derived compounds and extracts with immunomodulatory functions against influenza virus infection. Particular attention is given to their molecular targets within the TLR, RIG‐I, MAPK, and NF‐κB pathways, while also highlighting promising candidates and discussing the major challenges and prospects for developing plant‐based immunomodulators as novel influenza therapeutics.

## 2 Literature Search Strategy and Study Selection

A literature search was conducted in the Web of Science core collection, with cross‐checking performed in Scopus and PubMed using the same search terms, to identify studies on plant‐derived compounds as well as extracts modulating IAV replication via host signaling pathways. The search combined keywords for influenza (“influenza,” “influenza A virus,” “human influenza,” “H1N1,” “H3N2”), plant‐derived compounds with anti‐influenza activity (“plant‐derived compounds against influenza,” “anti‐influenza,” “plant derivative,” “botanical compounds,” “natural products,” “plant based antivirals,” “medicinal plants,” “phytochemical,” “herbal,” “plant compound,” “plant extract”), and relevant host signaling pathways (“MAPK,” “ERK,” “TLR,” “RLR”). The search covered publications from 2000 to 2025 and retrieved approximately 200 records. The review focused specifically on human IAV subtypes H1N1 and H3N2; studies on avian influenza and other non‐human strains were excluded. Studies reporting on Chinese or Japanese herbal formulations administered as decoctions, granules, or capsules were also excluded during selection.

## 3 Plant Compounds and Extracts Acting on IAV‐Induced Receptor‐Level Signaling

### 3.1 TLR Signaling Pathway

TLRs, a subset of pattern recognition receptors (PRRs), serve an essential function in the innate immune response through detecting and responding to invading pathogens. During IAV infection, TLR4 on the cell surface and TLR3, TLR7, and TLR8 in endosomes are key receptors studied for their role in defending against influenza. TLR3 recognizes double‐stranded RNA (dsRNA), while TLR7 as well as TLR8 detect single‐stranded RNA (ssRNA) (Lee et al. 2013; Kawasaki and Kawai 2014; de Marcken et al. 2019). Although IAV is a negative‐sense ssRNA virus, its replication cycle generates a variety of RNA species that engage multiple TLRs. During viral replication in the nucleus, complementary RNA and dsRNA intermediates can form, which are recognized by TLR3. In parallel, the genomic ssRNA and certain viral transcripts are sensed by TLR7 in endosomes (Diebold et al. 2004; Lund et al. 2004). Thus, TLR3, TLR7, and TLR8 contribute to innate immune recognition of IAV. TLR4, on the other hand, is activated not by direct viral components but by host‐derived damage‐associated molecular patterns such as oxidized phospholipids released during IAV‐induced oxidative stress (Olejnik et al. 2018). Notably, TLR3 serves as a key initiator of type I IFN responses, activating TANK (TRAF family member Associated NF‐κB Activator)‐binding kinase 1 (TBK1) through interaction with the TIR‐domain‐containing adaptor protein (TRIF), leading to phosphorylation of IFN regulatory factor 3 (IRF3) and induction of IFN‐β expression (Ascough et al. 2018). However, overexpression of IFN‐β during IAV infection may contribute to cytokine storm and severe lung damage. Autopsy reports from individuals with fatal IAV infections have revealed prolonged and elevated TLR4 and its adaptor myeloid differentiation primary response 88 (MyD88) expression in lung tissues. Thus, the TLR4/MyD88/NF‐κB axis represents a double‐edged sword, essential for initiating antiviral immunity but potentially detrimental if dysregulated (Paquette et al. 2012).

#### 3.1.1 Effect of Plant Compounds on TLR Activation Induced by IAV

Given these immune dynamics, plant‐derived compounds that target TLR pathways offer promising therapeutic potential. For example, quercetin, a flavonoid, treatment in a 16HBE (human bronchial epithelial cell line)–immune cell co‐culture model reduced H1N1‐induced cell death and activation of the TLR7/NF‐κB p65 axis (Figure 1, Table S1). Beyond protecting epithelial cells from virus‐induced mortality within 24–48 h, Api attenuated the upregulation of TLR7, MyD88, interleukin 1 receptor‐associated kinase 4 (IRAK4), TRAF6, and NF‐κB p65 transcripts and concurrently suppressed TLR7 and NF‐κB p65 protein expression in infected immune cells. These findings indicate modulation of IAV‐driven innate immune signaling, although it remains unclear whether this effect reflects reduced viral replication or direct interference with host signaling pathways. The study also did not directly quantify viral replication or identify molecular binding targets of quercetin. Furthermore, the absence of target‐binding assays or pathway‐specific perturbation experiments limits the mechanistic interpretation of quercetin's antiviral activity (Chen et al. 2012; Choi et al. 2017). Similarly, dihydromyricetin, a natural flavonoid, markedly inhibited TLR3 and its adaptor TRIF at the levels of gene and protein expression in IAV‐infected A549 cells (a human lung adenocarcinoma epithelial cell line), leading to decreased levels of pro‐inflammatory cytokines including CCL5, IFN‐γ–induced protein 10 (IP‐10), monokine induced by IFN‐γ and IFN‐β (MIG) (Figure 1, Table S1). This reduction in cytokine response may help prevent IAV‐induced inflammation. Additionally, dihydromyricetin has been shown to reduce H1N1 and H3N2‐induced plaque formation in a concentration‐dependent manner (IC_50_: 11.67–23.33 μM) and to target the PB2 subunit of IAV polymerase using a mini‐replicon assay as well as surface plasmon resonance, underscoring its dual activity, including both antiviral effect as well as anti‐inflammatory action. However, the precise binding site and functional relevance of this interaction were not validated through mutational or structural studies, and therefore, the detailed molecular mechanism remains to be fully elucidated (Tian et al. 2020). Epigallocatechin gallate (EGCG), another major flavonoid, primarily targets an early stage of the IAV subtype H1N1 and H3N2 replication cycle with the EC_50_ ranging from 5.7 to 17.3 μM in MDCK cells (Kim et al. 2013). Oral administration (10 mg/kg) of EGCG significantly decreased protein levels of TLR4 and NF‐κB in the BALB/c model of H9N2 swine IAV‐induced lung injury (Figure 1, Table S1). However, the study did not assess the IAV load in lung tissue, which limits the conclusions regarding its in vivo antiviral activity (Xu et al. 2017). Collectively, these findings suggest that the antiviral activities of these flavonoids may, at least in part, arise from their capacity to modulate TLR‐mediated signaling, thereby dampening excessive innate immune activation and inflammation during influenza virus infection.

**Figure 1:**
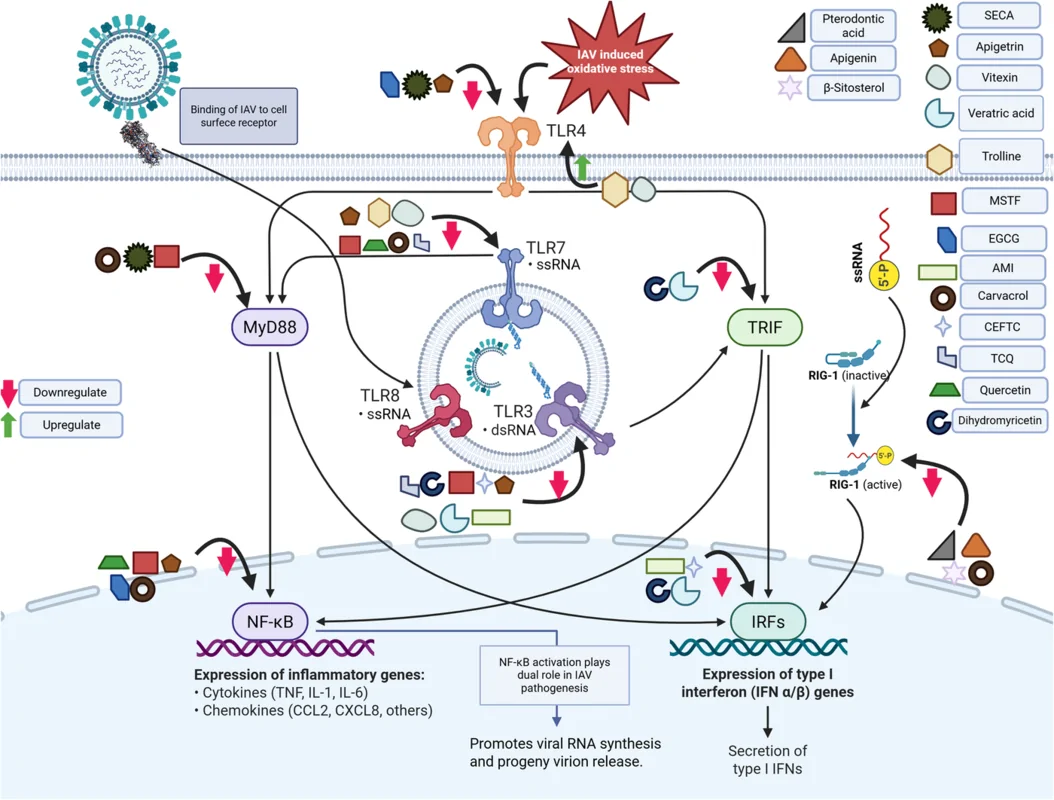
Illustration of the modulatory effects of plant‐derived compounds on IAV‐induced TLR and RIG‐I signaling pathways. The figure highlights how specific phytochemicals inhibit key molecules such as TLR3, TLR4, TLR7, MyD88, TRIF, RIG‐I, and IRF3, thereby suppressing downstream production of type I IFNs and pro‐inflammatory cytokines (created in https://BioRender.com). AMI, *Astragalus membranaceus* injection; CEFTC, crude extract of flowers of *Trollius chinensis*; EGCG, epigallocatechin gallate; IRFs, interferon regulatory factors; MSTF, *Mosla scabra* derived total flavonoids; MyD88, myeloid differentiation primary response 88; RIG‐I, retinoic acid inducible gene‐I; SECA, sesquiterpene‐rich extract of *Carpesium abrotanoides*; TCQ, 3,4,5‐Tri‐O‐caffeoylquinic; TRIF, TIR domain containing adapter inducing IFN‐β.

Curcumin, a polyphenolic compound derived from *Curcuma longa*, has demonstrated both direct antiviral and immunomodulatory activity against IAV, engaging TLR, MAPK, and NF‐κB signaling concurrently. In MDCK cells and a mouse model of IAV‐induced pneumonia, curcumin directly inactivated IAV virions and blocked viral adsorption (EC_50_ = 21.36 µg/mL), while separately suppressing IAV‐induced activation of TLR2, TLR4, and TLR7, p38/c‐Jun N‐terminal kinase (JNK) MAPK, and NF‐κB signaling. This dual action was accompanied by increased nuclear factor erythroid 2–related factor 2 (Nrf2), heme oxygenase‐1 (HO‐1), and IFN‐β expression, and by reduced lung viral titer, inflammatory cytokines, and improved survival and pulmonary histopathology in vivo, indicating that curcumin's antiviral effect is not solely attributable to immune signaling modulation (Chen et al. 2010; Chen et al. 2013; Dai et al. 2018; Han et al. 2018; Liew et al. 2025). In RAW264.7 cells (a murine macrophage‐like cell line), three major bioactive constituents derived from *Trollius chinensi*s, including vitexin, trolline, and veratric acid, demonstrated anti‐H1N1 activity by modulating TLR3, TLR7, and TLR4 (Figure 1, Table S1) (Shi et al. 2020). These compounds helped to reduce inflammation by lowering nitric oxide (NO), interleukin‐1 (IL‐1), IL‐6, and tumor necrosis factor‐α (TNF‐α) production while simultaneously enhancing IFN‐β expression to promote viral clearance. Veratric acid suppressed the TLR3 axis by downregulating TLR3, IRF3, TBK1, and TRIF, while vitexin exerted dual regulatory effects by inhibiting TLR3 and activating the TLR4 pathway. Similarly, trolline reduced TLR7 expression while activating the TLR4 pathway through upregulation of IκB kinase‐α (IKKα) and transforming growth factor‐β–activated kinase 1 (TAK1), collectively contributing to a balanced antiviral and anti‐inflammatory response. It is noteworthy that this study primarily assessed TLR‐related mRNA and protein expression in IAV‐infected macrophages, without directly quantifying viral replication. As a result, although the data suggest potential immunomodulatory activity during IAV infection, the precise mechanism, whether attributable to direct antiviral effects or indirect modulation of host pathways, remains unresolved. Moreover, the absence of in vivo studies evaluating these compounds needs to be addressed to further validate their efficacy. Thus, the reported “anti‐H1N1 activity” likely reflects immunomodulatory effects that create a less permissive environment for viral propagation, rather than a demonstrated reduction in viral load (Shi et al. 2020). Carvacrol, a phenolic compound derived from *Mosla chinensis* (Xiang ru), significantly downregulated TLR7, RIG‐I, MyD88, and NF‐κB protein expression in H1N1‐infected C57BL/6 mice (Figure 1, Table S1). Treatment with carvacrol (50 mg/kg) mitigated influenza‐induced body weight loss, reduced lung viral RNA load, and lowered the lung index. Histopathological examination further demonstrated that carvacrol protected against H1N1‐induced pulmonary injury by alleviating edema, hyperemia, and lymphocyte infiltration (Zheng et al. 2021). Additionally, 3,4,5‐tri‐O‐caffeoylquinic acid (TCQ), isolated from *Ilex pubescens*, significantly inhibited IAV subtype H1N1 infection in NCI‐H292 (a human epithelial cell line derived from lung mucoepidermoid carcinoma) cell culture at 200 μM, a concentration that exhibited minimal cytotoxicity. Kyoto Encyclopedia of Genes and Genomes (KEGG) pathway analysis revealed TCQ may inhibit both TLR3 and TLR7 pathways in IAV‐infected NCI‐H292 cells (Figure 1, Table S1), which resulted in reduced type I IFN expression and suppression of the Janus kinases–Signal transducers and activators of transcription (JAK‐STAT) signaling cascade to maintain balanced cytokine production. Nevertheless, the discussed studies in this section did not directly measure viral replication nor identify specific molecular targets of 3,4,5‐TCQA, limiting the mechanistic interpretation. Consequently, it remains unclear whether the observed antiviral effects are a result of direct viral inhibition or indirect immunomodulation. Further studies are required using a small animal model to validate the in vitro findings of the mentioned compounds, such as vitexin, trolline, veratric acid, as well as TCQ, and to address key pharmacological issues involving bioavailability, metabolism, toxicity, and so forth, of the plant compounds (Wang et al. 2024).

#### 3.1.2 Effect of Plant Extracts on TLR Activation Induced by IAV

The total flavonoids of *M. chinensis* (MSTF) significantly downregulated TLR3, TLR7, MyD88, TNF receptor‐associated factor 3 (TRAF3), and NF‐κB p65 expression in H1N1‐infected Institute of Cancer Research (ICR) mice models (Figure 1, Table S2). MSTF (288 or 576 mg/kg) treatment for 5 days through oral gavage alleviated lung pathology in mouse models of viral pneumonia, significantly reduced lung indices, and balanced pro‐inflammatory (IL‐6, TNF‐α, IFN‐γ) responses contributing to enhanced viral clearance and reduced lung injury (Zhang et al. 2018). However, lung viral titers were not assessed in this study, limiting direct assessment of antiviral efficacy. Further studies are needed to isolate and assess the effects of individual MSTF flavonoids. Similarly, intragastric administration (0.2 mg/g) of the crude extract of *T. chinensis* known as Chinese globe flower (CEFTC) for 7 days exhibited inhibitory effects on the TLR3–TBK1–IRF3 pathway by downregulating the expression of TLR3, TBK1, TAK‐1, and IRF3 in response to high‐dose IAV subtype H1N1 infection in ICR mice (Figure 1, Table S2) (Liang et al. 2023). Interestingly, this suppression was accompanied by an increase in IFN‐β secretion in mouse alveolar epithelial cells, suggesting that CEFTC may exert its antiviral effect through feedback regulation or other signaling pathways besides TLR3. This observation underscores the dynamic interplay between plant compounds and host immune responses (Liang et al. 2023).

*Astragalus membranaceus* (Huang Qi), a traditional Chinese medicinal herb, is derived from the dried roots of *Astragalus mongolicus* or *Astragalus membranaceus*. Injection of purified extracts of *A. membranaceus* (AMI) also demonstrated antiviral effects in RAW264.7 cells through suppression of the TLR3–TBK1–IRF3 signaling pathway (Figure 1, Table S2). It demonstrated antiviral activity against H1N1, as treatment with 9.5–92 mg/mL significantly alleviated virus‐induced inhibition of cell proliferation, with 9.5 mg/mL showing a statistically significant effect compared to the virus control. Correspondingly, AMI downregulated TLR3 and its downstream effectors at a concentration of 9.5 mg/mL, including TAK1, TBK1, IRF3, and IFN‐β, indicating inhibition of the innate antiviral axis. This effect may be associated with AMI's ability to alleviate virus‐induced G1 arrest and support cell cycle recovery, though the contribution of inflammation or cell death to this process was not clarified. AMI may further exert immunomodulatory effects through the TLR3–TAK1–NF‐κB pathway, although further investigation is warranted (Liang et al. 2019). In 16HBE cells, polysaccharides from *Isatis indigotica* (*Radix isatidis*) inhibited H1N1‐induced TLR3 activation and reduce the expression of IL‐6, IP‐10, MIG, and CCL‐5 triggered by A/PR/8/34 (H1N1) at multiple doses, with inhibition of progeny IAV subtype H1N1 and H3N2 observed at the concentration ranging from 4 to 20 mg/mL, suggesting a potential role in mitigating cytokine release and inflammation associated with lung injury (Figure 1, Table S2) (Li et al. 2017). These findings suggest significant therapeutic potential for plant‐derived extracts and compounds to fine‐tune TLR‐mediated innate immune responses, thereby offering dual benefits in controlling IAV replication and mitigating inflammatory damage.

### 3.2 Plant Compounds Interacting With IAV‐Induced RIG‐I Signaling Pathway

RIG‐I is a cytosolic PRR receptor involved in detecting viral RNA and initiating antiviral immune responses. Upon recognizing viral RNA, RIG‐I engages the mitochondrial adaptor protein, mitochondrial antiviral‐signaling protein (MAVS), triggering a series of signaling cascades that activate IRF3 and NF‐κB, leading to the production of type I and II IFNs and proinflammatory cytokines (Liu et al. 2017). Released IFNs bind to their respective receptors and activate IFN‐stimulated genes that restrict viral replication. In addition, RIG‐I activation promotes the release of chemokines that recruit immune cells to the infection site, thereby facilitating viral clearance. However, the role of RIG‐I in IAV infection is nuanced. While it drives protective antiviral responses, some studies suggest that RIG‐I‐mediated recruitment of monocyte‐derived dendritic cells may paradoxically enhance viral replication in IAV‐infected lungs, underscoring the complexity of its function (Pang et al. 2013). This section highlights plant‐derived compounds with demonstrated potential to modulate RIG‐I signaling during IAV infection.

Beyond promoting antiviral cytokine cascades, RIG‐I activation and its downstream signaling can also trigger cell death (Elion and Cook 2018). Apigenin is a flavonoid that was found to suppress RIG‐I activation and its downstream immune responses during IAV subtype H1N1 infection (Figure 1, Table S1) (Liu et al. 2008; Xu et al. 2020). In A549 cells, apigenin provided protection against cell death induced by high viral loads, likely mediated by reducing the production of IFNs (IFN‐β and IFN‐λ1) and proinflammatory cytokines (TNF‐α, IL‐6), as demonstrated by reverse transcription quantitative polymerase chain reaction (RT‐qPCR) assay (Xu et al. 2020). Mechanistically, apigenin disrupted RIG‐I–mediated signaling by promoting RIG‐I degradation. It interfered with the stabilizing interaction between RIG‐I and the chaperone protein Hsp90α, whose inhibition led to enhanced ubiquitination and proteasomal degradation of RIG‐I, a process further facilitated by increased binding to the E3 ligase RNF125 (Matsumiya et al. 2009; He et al. 2015; Mehmood et al. 2017). Importantly, apigenin did not significantly alter RIG‐I's interactions with other regulatory proteins such as USP3, USP21, or RNF122. Functionally, this reduction in RIG‐I levels diminishes the host cell's ability to sense viral RNA and mount type I IFN responses, thereby suppressing the antiviral signaling cascade. This highlights apigenin's potential as a modulator of excessive innate immune activation, which could be relevant in limiting virus‐induced inflammation (Xu et al. 2020). Apart from that, apigenin dose‐dependently decreased the expression of IAV proteins (NS1, NP, M1, and M2) as well as NP RNA levels in A549 cells. The study further suggested that apigenin may inhibit viral NA activity; however, the mechanistic evidence remains limited, as the study did not identify a definitive molecular binding target responsible for these effects. In addition to that, the relative contribution of direct viral inhibition versus host signaling modulation was not fully resolved (Matsumiya et al. 2009; He et al. 2015; Mehmood et al. 2017; Xu et al. 2020). Similarly, pterodontic acid, a triterpenoid compound (Figure 1, Table S1), exhibited subtype‐dependent antiviral activity against H1N1 as well as H3N2 (IC_50_: 9.47–37.14 μg/mL) and reduced viral propagation by inhibiting viral RNP export. The compound also reported to decrease RIG‐I expression in A549 cells, thereby suppressing induction of the NF‐κB pathway and contributing to reduced inflammatory signaling during IAV infection. This results in a significant reduction of the expression of macrophage inflammatory protein‐1 β, IL‐6, monocyte chemoattractant protein‐1 (MCP‐1), and IP‐10 (IFNγ‐induced protein 10) induced by H1N1, revealed by RT‐qPCR (Guan et al. 2017; Wang et al. 2019).

β‐sitosterol, a phytosterol found in various traditional Chinese herbal plants, is reported to suppress RIG‐I signaling pathways, thereby attenuating the IAV‐induced production of IFNs (IFN‐β, IFN‐γ, and IFN‐λ1) and proinflammatory cytokines (Figure 1, Table S1) (Jiang et al. 2011). Previous studies have demonstrated the link between RIG‐I expression and activation of the STAT1 pathway. In IAV‐infected and IFN‐stimulated cells, β‐sitosterol disrupted this axis by interfering with RIG‐I‐mediated STAT1 activation, leading to reduced expression of key inflammatory mediators such as IL‐6, IL‐8, TNF‐α, and IP‐10 (Figure 1, Table S1). Furthermore, β‐sitosterol inhibited the induction of downstream effectors, including NF‐κB and p38 MAPK, in both IAV‐infected and viral RNA‐transfected cells (Zhou et al. 2020). These findings suggest that β‐sitosterol antagonizes the RIG‐I signaling cascade, effectively dampening excessive inflammatory responses during IAV infection. However, the study did not include direct viral replication assays to measure the viral proliferation.

It is important to note that many studies investigating these phytochemicals have focused primarily on host signaling and cytokine modulation rather than directly assessing infectious viral titers. Consequently, it remains possible that immune dampening could increase replication in some contexts; future studies should pair cytokine profiling with plaque/TCID_50_ assays. However, certain pathways, such as the RIG‐I/IFN signaling axis, play a crucial role in restricting viral replication and dissemination. Therefore, suppression of these responses may alleviate inflammation but could inadvertently promote enhanced viral replication. Taken together, plant‐derived compounds such as apigenin, pterodontic acid, and β‐sitosterol exhibit potential in modulating the RIG‐I signaling to reduce IAV‐induced inflammation. Their targeted action on innate immune signaling pathways highlights promising avenues for the development of immunomodulatory therapeutics, which can be used with antiviral drugs aimed at balancing immune activation and pathology. However, a common and major limitation of these studies is not identifying specific molecular targets or experimentally separate direct effects on viral processes from indirect effects mediated via host signaling, leaving the precise mechanism unresolved.

## 4 Interaction of Plant Compounds and Plant Extracts With IAV‐Induced Downstream Effector Signaling Pathway

### 4.1 MAPK Signaling Pathway

The MAPK pathway, comprising extracellular signal‐regulated kinase (ERK), p38 MAPK, and JNK, plays a dual role during IAV infection. These pathways regulate host cell signaling and contribute to both viral replication and immune response activation, highlighting their complex involvement in IAV pathogenesis (James et al. 2015; Choi et al. 2020).

#### 4.1.1 Blocking vRNP Nuclear Export Through ERK Inhibition by Plant Compounds

Various studies have underscored the crucial role of ERK phosphorylation in supporting IAV replication (Pleschka 2008). As a component of the MAPK signaling cascade, ERK is a ubiquitously expressed intracellular kinase involved in essential cellular functions, encompassing both proliferation and cellular differentiation. Notably, independent studies have reported that ERK activation facilitates IAV infection (Pleschka et al. 2001; Gangehei et al. 2010). Specifically, ERK1 signaling is required for the nuclear export of vRNP complexes, a critical step in the IAV replication cycle (Pleschka et al. 2001). At the same time, ERK activation plays an essential role in antiviral defense by regulating cytokine production, immune cell activation, and phagocytic activity; therefore, its inhibition, while restricting viral replication, may also dampen protective immune functions (Lucas et al. 2022). ERK activation is primarily regulated via the RAS/RAF/MEK pathway and can also be modulated by protein kinase C‐α (PKC‐α). Upon infection, IAV induces PKC‐α activation, which enhances ERK phosphorylation and promotes nuclear export of vRNP, thereby facilitating efficient viral propagation (Wen‐Sheng and Jun‐Ming 2005; Marjuki et al. 2006). During replication, helical vRNPs are formed by packaging viral RNAs within the host cell nucleus and consequently assembled with structural proteins in the cytosol (Cros and Palese 2003). Several natural compounds have been found to interfere with this ERK‐driven process. Berberine (BBR), an isoquinoline alkaloid, treatment with 25 μM was shown to block H1N1 (A/PR8/34) growth in macrophage and epithelial cells (RAW 264.7, A549) but not in MDCK cells (Cecil et al. 2011). Mechanistic studies suggested BBR inhibits IAV replication by blocking ERK1‐mediated vRNP nuclear export (Figure 2, Table S1), indicating that its antiviral effect stems from disrupting host cell signaling pathways critical to viral propagation (Botwina et al. 2020). Again, no direct binding of BBR to ERK (or any viral protein) was shown; the conclusions are instead inferred from pathway‐level evidence and comparative interpretation (Cecil et al. 2011; Botwina et al. 2020). BBR has been shown to suppress ERK activation and downstream pro‐inflammatory cytokine production in macrophages, even in the absence of IAV infection, but in response to inflammatory stimuli, suggesting potential immunomodulatory consequences for uninfected host cells (Jeong et al. 2009; Wang et al. 2020). Similarly, patchouli alcohol (PA), a sesquiterpene derived from *Pogostemonis herba*, had clear anti‐influenza activity (IC_50_ ≈ 2–6 μg/mL for multiple H1N1 strains) through plaque reduction when pre‐incubated with virus. PA significantly suppressed ERK1/2 activation in IAV‐infected MDCK cells (Figure 2), with minimal influence on NF‐κB signaling. This suggests PA primarily exerts its antiviral effects by targeting the ERK/MAPK axis, rather than general inflammatory pathways. However, the study does not identify any specific molecular target for PA. The target of PA was only inferred indirectly by blocking the virus replication cycle, which normally triggers PI3K/Akt and ERK pathways (Yu et al. 2019). Furthermore, pterodontic acid was found to suppress ERK phosphorylation in IAV‐infected A549 cells, disrupting the nuclear export of vRNPs (Figure 2, Table S1). However, since this was only observed during viral infection, it is unclear whether ERK is a direct target or if its phosphorylation is reduced indirectly by the antiviral effect (Wang et al. 2019). Consistent with these findings, β‐sitosterol, previously discussed for its effects on RIG‐I, was also shown to inhibit ERK1/2 activation in the IAV‐infected mouse lung tissue, suggesting a broader immunomodulatory role that includes reduction of ERK‐mediated viral replication. However, the lung viral titer was not quantified in this study after β‐sitosterol treatment (Zhou et al. 2020). The reduction in phosphorylated ERK1/2 observed in vivo may reflect the immune‐regulating action of β‐sitosterol.

**Figure 2:**
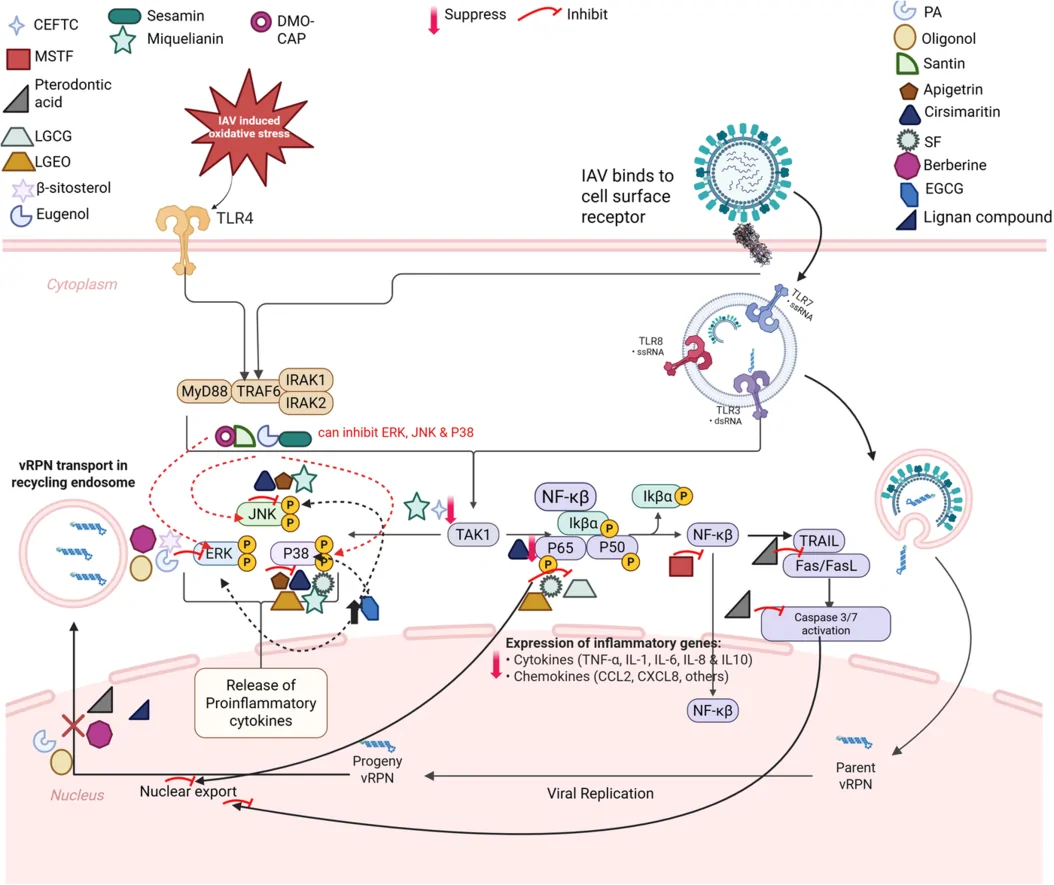
Schematic representation of how plant‐derived compounds modulate the MAPK and NF‐κB signaling pathways activated by infection owing to IAV replication. The figure illustrates the inhibitory effects of phytochemicals on key kinases such as p38, JNK, ERK1/2, TAK1, P65 and on the translocation and activation of NF‐κB, ultimately suppressing the expression of inflammatory cytokines and viral replication during IAV infection (created in https://BioRender.com). CEFTC, crude extract of flowers of *Trollius chinensis*; DMO‐CAP, 6‐demethoxy‐4′‐O‐methylcapillarisin; EGCG, Epigallocatechin gallate; ERK, extracellular signal regulated kinase; IRAK, interleukin receptor‐associated kinase 1; IκBα, inhibitor of nuclear factor κB; JNK, c‐Jun N‐terminal kinase; *LGCG*, lignan glycosides compound from *Calotropis gigantea*; *LGEO*, lignan glycoside [(+)‐pinoresinol‐O‐β‐D‐glucopyranoside] isolated from *Eucommia ulmoides* Oliver; MSTF, *Mosla scabra* derived total flavonoids; MyD88, myeloid differentiation primary response 88; NF‐κB, nuclear factor‐κB; PA, patchouli alcohol; SF, sesquiterpene fraction from *Laggera pterodonta*; TAK1, transforming growth factor β‐activated kinase 1; TRAF6, TNF receptor associated factor 6; TRAIL, tumor necrosis factor (TNF)‐related apoptosis‐inducing ligand.

#### 4.1.2 Plant Compounds Causing Broad MAPK Suppression and Synergistic NF‐κB Inhibition

IAV infection activates multiple branches of the MAPK signaling pathway, including ERK, p38 MAPK, and stress‐activated protein kinase/JNK (Zhong, Wang, Ma, et al. 2019). Activation of these MAPK pathways, along with NF‐κB phosphorylation, has been linked to increased expression of proinflammatory cytokines such as IL‐8, IL‐1β, TNF‐α, as well as the anti‐inflammatory cytokine IL‐10 following IAV infection (Chan et al. 2005; Liou and Huang 2017). Santin, a flavonoid isolated from *Artemisia rupestris*, suppressed IAV M2 protein and RNA expression in a dose‐dependent manner (IC_50_: 27.6–37.2 μM), with immunofluorescence, RT‐qPCR, and western blotting (WB) analyses collectively confirming its antiviral activity against influenza in MDCK and THP‐1 cells (human monocytic cell line). Moreover, santin significantly reduced IAV‐induced phosphorylation of ERK, p38, and JNK in THP‐1 cells infected with IAV, which might contribute to effective suppression of viral infection (Zhong, Wang, Yan, et al. 2019). IAV infection also elevates proinflammatory cytokines like IL‐1β, IL‐6, IL‐8, and TNF‐α, all of which were downregulated by santin treatment in THP‐1 cells (Zhong, Wang, Yan, et al. 2019). Similarly, sesamin, a lignan compound, was shown to suppress H1N1 IAV infection in peripheral blood mononuclear cells (PBMCs) by reducing NA activity. It also inhibits MAPK signaling, including decreased phosphorylated of JNK, p38, and ERK1/2 in H1N1‐infected PBMCs. This inhibition correlated with reduced proinflammatory cytokines (IL‐1β and TNF‐α) and increased levels of the immune‐supportive cytokine IL‐2. However, it should be noted that this study did not directly quantify IAV replication and therefore the observed immunomodulatory and NA inhibitory effects cannot be unequivocally attributed to a reduction in viral replication (Fanhchaksai et al. 2016). Another plant‐derived phenolic compound, eugenol, markedly reduced IAV subtype H1N1 and H3N2 replication at concentrations ranging from 0.625 to 5 µg/mL. It was found to block IAV‐induced activation of ERK1/2, p38, and NF‐κB pathways in A549 cells (Dai et al. 2013). Additional in vivo investigations are also required to comprehensively clarify the antiviral and immunomodulatory mechanisms of santin, sesamin, and eugenol (Figure 2, Table S1).

Two independent studies reported that the plant‐derived compounds apigetrin (in A549 cells) and cirsimaritin (CST) (in THP‐1 cells) inhibited IAV‐induced phosphorylation of JNK and p38 MAPK (Figure 2, Table S1) (Yan et al. 2018; He et al. 2023). Plaque assay data demonstrated that apigetrin significantly inhibited replication of IAV subtype H1N1 (EC_50_ = 0.19 μg/mL) across concentrations ranging from 0.25 to 2 μg/mL, with the antiviral effect observed during the early post‐infection period (1–5 h p.i.). Apigetrin also reduced lung index, IAV replication, and multiple pro‐inflammatory mediators (IL‐6, IL‐8, TNF‐α, IL‐1β), while high‐dose treatment prolonged survival and alleviated lung pathology in infected BALB/c mice (He et al. 2023). Similarly, CST efficiently inhibited multiple IAV subtypes (H1N1 and H3N2), with IC_50_ values ranging from 5.8 to 11.1 μg/mL in MDCK cells. WB and RT‐qPCR analyses demonstrated that CST treatment dose‐dependently lowered viral titers and reduced IAV M2 protein and RNA levels in both infected MDCK and THP‐1 cells. The absence of mouse models in these studies limits the translational relevance of the reported antiviral effects (Yan et al. 2018). Additionally, the lignan glycoside (+)‐pinoresinol‐O‐β‐D‐glucopyranoside (LGEO), isolated from *Eucommia ulmoides* (Hardy rubber tree), was found to suppress IAV‐induced p38 MAPK phosphorylation in A549 cells. This suppression appears to underlie its antiviral activity, as LGEO did not inhibit p38 MAPK activation triggered by poly (I:C) (Figure 2, Table S1) (Li et al. 2019). Notably, although the LGEO inhibited p38 MAPK activation, it lacked antiviral activity against certain IAV subtypes such as H3N2, H9N2, and influenza B virus. But this lignan glycoside diminished the CPE effects caused by two H1N1 strains (A/PR/8/34 and A/Guangzhou/GIRD07/09) revealed by the CPE reduction assay. Miquelianin, a flavonoid, showed inhibition of IAV H1N1 subtype replication with EC_50_ values of 107.8 µM. Based on KEGG pathway analysis, miquelianin is suggested to reduce IAV‐induced inflammation by inhibiting TAK1 activation (Li et al. 2025). This suppression downregulates downstream JNK1 and p38 MAPK signaling, leading to decreased proinflammatory cytokine production and reduced tissue inflammation in IAV‐infected BALB/c mice (Figure 2, Table S1). Nevertheless, confirmation using TAK1‐deficient models or specific inhibitors is needed to substantiate the mechanistic role of TAK1 in IAV infection (Li et al. 2025).

Notably, there is a lack of investigation into the pharmacokinetic properties and bioavailability of studied compounds within IAV‐infected lung tissue, raising uncertainty as to whether the effective concentrations observed in vitro are achievable in vivo. The absence of these data further limits the translational relevance of the compounds.

#### 4.1.3 Plant Compounds Causing MAPK Activation‐Driven Induction of Antiviral Effectors

In a mechanistically distinct pattern, EGCG and DMO‐CAP activate rather than inhibit MAPK cascades to enhance antiviral host programs, illustrating that the same kinase cascade can serve opposing outcomes depending on which downstream target is engaged. Although EGCG does not interfere with HA or polymerase activity, it compromises viral membrane integrity, preventing hemifusion with host membranes and thereby blocking viral internalization after attachment (Kim et al. 2013; Stannard et al. 2023). EGCG also significantly increased both mRNA and protein levels of human β‐defensin 3 in the bronchial epithelial cell line (BEAS‐2B), highlighting its potential to enhance innate antiviral defense. EGCG inhibits H1N1 IAV by inducing β‐defensin 3 expression through activation of the p38 MAPK, ERK, and JNK pathways (Figure 2, Table S1) (Mou et al. 2020). Additionally, EGCG boosts IFN‐λ2 expression via p38 MAPK activation, which further suppresses H1N1 replication in BEAS‐2B cells (Zhu et al. 2020). Elevated expression of HO‐1 enhances the IFN response and stimulates IFN‐regulated genes, contributing to its strong anti‐influenza effects (Zhong, Wang, Ma, et al. 2019). Mechanistically, DMO‐CAP activates p38, JNK, and ERK MAPK pathways (Figure 2, Table S1), leading to induction of the Nrf2/HO‐1 axis. Elevated expression of HO‐1 enhances the IFN response and stimulates IFN‐regulated genes, contributing to its strong anti‐influenza effects (Zhong, Wang, Ma, et al. 2019). Further studies, including synergy study and mice model, are needed to explore additional mechanisms by which DMO‐CAP inhibits IAV replication. Interestingly, while several phytochemicals, such as BBR, inhibit MAPK/ERK signaling to block vRNP nuclear export and suppress IAV replication, others, including EGCG and DMO‐CAP, activate MAPK cascades to enhance antiviral defenses through upregulation of host immune pathways. Thus, opposing modulation of the same pathway can confer antiviral benefits via distinct mechanisms, either by disrupting viral export or by amplifying host antiviral responses (Zhong, Wang, Ma, et al. 2019; Botwina et al. 2020; Mou et al. 2020). Together, these findings underscore the potential of plant‐derived flavonoids as modulators of MAPK signaling to combat IAV infection, offering promising avenues for the development of multifunctional phytotherapeutics that balance viral suppression with immune regulation.

#### 4.1.4 Effect of Plant Extracts Against IAV‐Induced MAPK Signaling

Oligonol, a polyphenol mixture, was reported to inhibit ERK phosphorylation during IAV subtype H3N2 infection (Kundu et al. 2009; Gangehei et al. 2010). Oligonol's mechanism might include the interference with the RAS/RAF/MEK pathway and/or PKC activation (Table S2). Moreover, some findings suggest it may interfere with the NS2 protein, which also mediates vRNP export, though this requires further validation (O'Neill et al. 1998; Gangehei et al. 2010). Additionally, a sesquiterpene fraction (SF) from *Laggera pterodonta* was shown to suppress IAV‐triggered p38 MAPK phosphorylation and release of cytokines in A549 cells (Figure 2, Table S2). CPE assays showed that SF inhibited IAV H1N1 and H3N2 subtypes in MDCK cells and displayed strong antiviral activity during the early stages of infection (0–6 h), affecting viral entry and adsorption (Wang et al. 2017). Studies on MSTF (Yu et al. 2016; Cai and Zhang 2022) and total flavones from *Abelmoschus manihot* (TFA) (Table S2) also showed antiviral effects against IAV infection by suppressing MAPK signaling and reducing inflammatory cytokine secretion in infected mice (Figure 2, Table S2) (Gao et al. 2022). MSTF (50–200 mg/kg) dose‐dependently reduced inflammatory cell infiltration, lung index, and viral load in ICR mice, with 200 mg/kg showing greater efficacy than amantadine, indicating protection against IAV‐induced lung injury and replication (Cai and Zhang 2022). Likewise, TFA inhibited IAV replication (IC_50_ = 5.36 μg/mL) and dose‐dependently reduced viral RNA and pro‐inflammatory cytokine mRNAs (IL‐1β, IL‐6, TNF‐α) in H1N1‐infected MDCK cells. In TFA‐treated C57BL/6 mice, viral RNA levels were markedly reduced by day 3 post‐infection (Gao et al. 2022). However, further research is required to clarify the specific actions of individual components within these flavonoid mixtures. In parallel, crude phenylethanoid glycosides extracted from *Ligustrum purpurascens* (Table S2) also showed antiviral effects by promoting endogenous IFN‐γ production in C57BL/6J mice. In vitro, PhG treatment (0.5 mg/mL) significantly suppressed H1N1 infection in MDCK cells, while in vivo studies demonstrated improved survival and reduced lung pathology, edema, and inflammation in infected C57BL/6 J mice. These benefits were associated with enhanced endogenous IFN‐γ production, although the detailed signaling mechanisms remain to be clarified (Song et al. 2012; Hu et al. 2016).

The studies demonstrate that the plant compounds, as well as plant extracts, suppress MAPK and NF‐κB signaling during influenza virus infection. Another common limitation across studies of phytochemicals against IAV is the lack of assessment of compound bioavailability in the respiratory tract, the primary site of infection. While in vitro cell lines (MDCK, A549) demonstrate antiviral activity, it is unclear whether the effective concentrations are achievable in vivo. Future studies employing pharmacokinetic measurements in lung tissue, bronchoalveolar lavage, or primary airway epithelial cultures are needed to validate translational relevance.

### 4.2 NF‐κB Signaling Pathway

NF‐κB is a widely expressed transcription factor that regulates the expression of over 100 host genes associated with immune and inflammatory responses (Garoufalis et al. 1994; Pahl and Baeuerle 1995; Ronni et al. 1997; Flory et al. 2000). During IAV infection, NF‐κB activation enhances the transcription of key viral genes, including HA, NP, and M1, which are essential for efficient viral replication. This reflects a dual role for NF‐κB in IAV infection. Beyond driving inflammatory gene expression, NF‐κB signaling is also directly required for viral genomic RNA (vRNA) synthesis, so its inhibition can exert antiviral effects independent of its immunomodulatory role (Kumar et al. 2008). Notably, modulation of NF‐κB signaling in vitro and in vivo has been shown to balance the production of proinflammatory cytokines such as IL‐6, IL‐8, MCP‐1, and CCL‐5, thereby mitigating the risk of cytokine storm and virus‐induced lung injury (Pinto et al. 2011). Under basal conditions, NF‐κB is sequestered in the cytoplasm as an inactive complex, primarily composed of p50 and p65 subunits bound to inhibitory proteins from the IκB family, notably IκBα. This interaction prevents nuclear translocation of NF‐κB. Upon IAV infection, IκB is phosphorylated and degraded, freeing NF‐κB to translocate into the nucleus and activate transcription of genes involved in inflammation and antiviral defense (Zhong et al. 1998).

#### 4.2.1 Modulation of IAV‐Induced NF‐κB Signaling by Plant Compounds

CST, mentioned in the earlier section, has demonstrated potent anti‐influenza activity through modulation of the NF‐κB signaling pathway. In IAV‐infected THP‐1 cells, CST treatment significantly reduced the expression and nuclear phosphorylation of the NF‐κB p65 subunit (Figure 2, Table S1), leading to suppressed production of proinflammatory cytokines such as TNF‐α, IL‐1β, and IL‐8, along with decreased expression of cyclooxygenase‐2 (COX‐2) (Yan et al. 2018). This dual effect, viral replication inhibition and attenuation of the host inflammatory response, positions CST as a promising therapeutic candidate for IAV infection. IAV infection triggers the NF‐κB signaling pathway, which induces the expression of pro‐apoptotic factors such as TNF‐related apoptosis‐inducing ligand (TRAIL) and Fas/Fas ligand (Fas/FasL). These molecules facilitate caspase‐mediated nuclear export of vRNP complexes, a critical step in the IAV life cycle (Wurzer et al. 2003, 2004). Pterodontic acid has been shown to inhibit TRAIL and FasL expression (Figure 2), leading to downregulation of caspase‐3/7 activity and subsequent impairment of nuclear export of vRNPs, thereby suppressing viral replication (Guan et al. 2017; Wang et al. 2019). Similarly, lignan glycosides from *E. ulmoides* have demonstrated the ability to block IAV‐induced nuclear translocation of the NF‐κB p65 subunit (Figure 2, Table S1). By disrupting this key signaling event, these compounds are speculated to inhibit the transcription of viral and host inflammatory genes and impair nuclear export of vRNPs (Li et al. 2019). Apart from previously mentioned antiviral activity, curcumin was also shown to inhibit NF‐κB activation specifically in IAV‐infected macrophages, reducing IκBα phosphorylation and downstream pro‐inflammatory cytokine production, further supporting a role for curcumin in dampening IAV‐induced innate immune signaling independent of any direct antiviral effect in that model (Xu and Liu 2017). Andrographolide, the primary bioactive diterpenoid of *Andrographis paniculata*, has been shown to modulate IAV‐induced NF‐κB signaling. In a lethal H1N1 (PR8) mouse model, andrographolide treatment increased survival, reduced lung pathology, and decreased both viral loads and inflammatory cytokine expression, with mechanistic analysis implicating the NF‐κB signaling pathways in this effect. Notably, combining andrographolide with the viral entry inhibitor CL‐385319 produced a greater survival benefit than either compound alone, suggesting that andrographolide's immunomodulatory activity via NF‐κB signaling may complement, rather than substitute for, direct antiviral strategies targeting IAV entry (Ding et al. 2017). While these findings highlight novel therapeutic mechanisms, the precise molecular targets and signaling intermediates involved require further elucidation to fully validate their antiviral potential. Further clarification of these mechanisms could be achieved through time‐of‐addition and confocal imaging assays to determine the stage of vRNP inhibition, as well as NF‐κB reporter assays to confirm transcriptional effects. Complementary use of caspase inhibition or knockdown strategies may help distinguish NF‐κB–mediated actions from downstream TRAIL/FasL–caspase signaling. Such approaches would refine understanding of the molecular targets underlying the antiviral activity of these phytochemicals.

While the studies provide valuable insights into the antiviral and immunomodulatory effects of phytochemicals against IAV, it remains largely correlative. The mechanistic basis of action of the phytochemicals is not fully understood. Moreover, molecular binding targets are not identified, limiting mechanistic depth. The reliance on in vitro models without assessment of respiratory tract bioavailability further restricts physiological relevance. Finally, despite proposing phytochemicals as adjunctive therapies, the study lacks evaluation of synergistic interactions with approved antivirals and provides minimal discussion of clinical translation.

#### 4.2.2 Modulation of IAV‐Induced NF‐κB Signaling by Plant Extracts

CEFTC, a plant‐derived extract, may exert immunomodulatory response against IAV infection via inhibition of the TAK1‐NF‐κB signaling cascade, as revealed by WB analysis of infected lung tissue samples from mice (Figure 2, Table S2). In the CEFTC‐treated group, the lung index was significantly lowered, and lung pathology was notably alleviated compared with the infected controls. However, further mechanistic studies are warranted to confirm this mode of action of the individual compound from the CEFTC (Liang et al. 2023). Similarly, MSTF has been shown to alleviate IAV‐induced acute lung injury in murine models by downregulating NF‐κB activation through the TLR7 and RIG‐I signaling pathways (Figure 2, Table S2). MSTF treatment lowered serum IL‐6, TNF‐α, and IL‐1β levels while elevating IFN‐α, indicative of an anti‐inflammatory and immunoregulatory effect. Nonetheless, the viral load in lung tissues was not assessed, leaving the relationship between immune modulation and antiviral efficacy unresolved (Yu et al. 2016). These results highlight the therapeutic promise of flavonoid‐rich plant extracts in modulating host inflammatory responses induced by IAV infection. Additionally, previously mentioned SF from *L. pterodonta* also exhibited the ability to modulate IAV‐induced nuclear translocation of the NF‐κB p65 subunit, which suppresses the excessive release of inflammatory cytokines and chemokines (Wang et al. 2017) (Figure 2).

These findings highlight the multifaceted role of NF‐κB signaling in coordinating both viral replication and host inflammatory responses during IAV infection, establishing it as a pivotal target for therapeutic intervention. Plant‐derived compounds, including CST, pterodontic acid, MSTF, lignan glycosides, and sesquiterpene derivatives, exemplify how natural products can modulate aberrant NF‐κB activation to restore immune balance and mitigate disease pathology (Figure 2, Tables S1 and S2). Further mechanistic and in vivo investigations are warranted to validate these pathways and to evaluate the translational potential of these natural NF‐κB modulators in the management of influenza virus infection.

## 5 Linking Immune Signaling Modulation to Infection Outcome

Across the TLR, RIG‐I, MAPK, and NF‐κB pathways discussed above, the strength of evidence connecting a signaling change to a real infection outcome varies considerably, and this variability is itself an important finding of this review. Among individual compounds, the link is well supported in some cases, such as apigetrin (IC_50_: 5.8–11.1 μg/mL) and dihydromyricetin (IC_50_: 11.67–23.33 μM), each paired with pathway‐level suppression with dose‐dependent reductions in viral titer, giving evidence that the signaling change and the antiviral effect are mechanistically connected. In many other cases, however, this connection remains inferred rather than demonstrated: several compounds reviewed here (e.g., quercetin, vitexin, trolline, veratric acid) were shown to suppress TLR‐ or MAPK‐associated markers at the transcript or protein level, without any accompanying measurement of viral load, survival, or lung pathology. In these instances, it is difficult to determine whether the reported activity reflects a direct antiviral effect or an indirect immunomodulatory effect that renders the cellular environment less permissive to viral replication. Among plant extracts, a similarly mixed picture emerges where MSTF (200 mg/kg) reduced lung viral load and inflammatory infiltration in mice more effectively than amantadine, and TFA (IC_50_ = 5.36 μg/mL) both suppressed MAPK‐driven cytokine induction and produced a measurable drop in viral RNA by Day 3 post‐infection in vivo, both examples where signaling suppression and infection outcome were assessed together. This gap is compounded by a broader translational limitation noted throughout the literature: most effective concentrations are established in vitro (MDCK, A549), with little pharmacokinetic data confirming that comparable concentrations are achievable in lung tissue in vivo. This represents a genuine and fixable gap in the field; future work pairing mechanistic signaling data with functional virological and pathological endpoints will substantially strengthen the mechanistic evidence linking phytocompounds to their proposed roles as anti‐influenza immunomodulators.

## 6 Pathway Convergence and Cross‐Talk in Phytocompound‐Mediated Anti‐IAV Signaling

Throughout this review, the immunomodulatory effects of phytocompounds have been discussed across individual pathway sections, including TLR, RIG I, MAPK, and NF κB, yet the experimental findings consistently show interconnected activity spanning all four cascades rather than acting within isolated pathways. Both TLR3/7 (via MyD88/TRIF) and RIG‐I/MAVS signaling independently converge on TRAF6, which activates TAK1. It then bifurcates simultaneously into the IKK–IκBα–NF‐κB axis and the MAPK cascades (JNK, p38, ERK), while TBK1, recruited by both TRIF and MAVS, converges on IRF3 to drive type I IFN production. These shared signaling components mean that several phytocompounds reviewed here may engage multiple pathways through a single molecular target. Miquelianin suppressed IAV‐induced inflammation by inhibiting TAK1–JNK1–p38 signaling, simultaneously attenuating both NF‐κB and MAPK from a single convergence point that is likely shared by TLR and RIG‐I signaling pathways (Li et al. 2025). Similarly, carvacrol concurrently suppressed TLR7, RIG‐I, MyD88, and NF‐κB p65—illustrating that cross‐pathway activity at shared downstream effector is a consistent property of these compounds (Shi et al. 2020; Zheng et al. 2021).

The MAPK pathway exhibits substantive bidirectional cross‐talk with both NF‐κB and the IFN regulatory axis. JNK and p38 activation produce the activator protein 1 (AP‐1) transcription factor complex, which cooperates directly with NF‐κB on cytokine gene promoters during IAV infection (Yu et al. 2020). Compounds suppressing both cascades concurrently, including santin (ERK/p38/JNK and NF‐κB), sesamin (JNK/p38/ERK), and eugenol (ERK1/2, p38, and NF‐κB), achieve synergistic cytokine suppression that cannot be predicted from either pathway alone (Dai et al. 2013; Zhong, Wang, Ma, et al. 2019). Beyond NF‐κB, the MAPK and IFN arms are cross‐linked through MAPK phosphatase 5 (MKP5), which is induced by IAV infection in an NS1‐dependent manner and directly dephosphorylates IRF3, suppressing type I IFN production. MKP5‐deficient mice showed enhanced IRF3 activation and significantly greater resistance to H1N1 virus challenge (James et al. 2015). This means BBR's inhibition of ERK1‐mediated vRNP nuclear export and PA's ERK/MAPK suppression may indirectly modulate IFN‐β release through the MKP5–IRF3 axis, which is unaddressed in either study (Yu et al. 2019; Botwina et al. 2020). Notably, the apparently paradoxical observation that ERK1/2 inhibition (by PA), ERK1 inhibition (by BBR), and ERK activation (by DMO‐CAP) both confer antiviral activity is explained by ERK inhibition disrupting pro‐viral vRNP export, while ERK activation promotes antiviral IFN induction, both mediated by the ERK (Zhong, Wang, Ma, et al. 2019).

Simultaneous suppression of RIG‐I‐mediated STAT1 activation, NF‐κB, and p38 MAPK by β‐Sitosterol disrupts the positive IFN feedback loop in which type I IFN upregulates RIG‐I expression. This disruption prevents the progressive inflammatory amplification that drives cytokine hyperinduction and acute lung injury (Zhou et al. 2020). These examples, together with the evidence for TAK1‐mediated PRR convergence, MAPK–AP‐1–NF‐κB transcriptional coupling, and MKP5‐mediated MAPK–IRF3 cross‐regulation, collectively demonstrate that the phytocompounds reviewed here function as modulators of an integrated innate immune signaling network rather than selective inhibitors of discrete cascades. Future mechanistic studies should incorporate cross‐pathway experimental analyses, including pathway‐specific knockout or knockdown models (e.g., TAK1‐deficient or TRAF6‐silenced cells), selective pharmacological inhibitors to dissect individual signaling branches, co‐reporter assays measuring NF‐κB and AP‐1 transcriptional activity simultaneously, and proteomic profiling to capture network‐wide kinase activity to fully characterize the network‐level pharmacology of these natural compounds.

## 7 Clinical Translation Barriers of Phytocompounds and Plant Extracts as Anti‐IAV Therapeutics

Despite compelling preclinical evidence that phytocompounds discussed in this review modulate TLR, RIG‐I, NF‐κB, and MAPK signaling during IAV infection, none have progressed to clinical approval as an anti‐influenza therapeutic. This gap reflects deep‐rooted pharmacological barriers across interconnected domains of undefined molecular target, bioavailability, metabolism, and toxicity.

Poor oral bioavailability is a translational barrier across all chemical classes reviewed here. Flavonoids are characterized by low aqueous solubility, chemical instability at intestinal pH, and extensive presystemic metabolism. Human intervention studies report that only 3%–17% of an administered quercetin dose is absorbed, and peak plasma concentrations following typical ingestion rarely exceed 1–2 μM which is far below the 10–50 μM concentrations used in the in vitro IAV models (Hollman et al. 1995; Olthof et al. 2000). EGCG, discussed here as a TLR4/NF‐κB modulator and MAPK activator, undergoes rapid auto‐oxidation and epimerisation at physiological pH, resulting in extensive gastrointestinal degradation before absorption; clinically achievable plasma concentrations (7.5 µM) from oral dosing fall well short of reported EC_50_ values in MDCK and A549 cells (Yang et al. 2008). In case of BBR, despite its membrane‐interactive quaternary ammonium structure, P‐glycoprotein‐mediated efflux at the intestinal epithelium, poor aqueous solubility (~2 mg/mL), and hepatobiliary re‐excretion collectively limit oral bioavailability to below 5% (Battu et al. 2010; Liu et al. 2016). β‐Sitosterol exhibits extremely poor oral bioavailability (~0.41%), with more than 99.5% of the administered dose failing to enter systemic circulation. This was demonstrated in healthy human subjects using a ^14^C‐labeled tracer approach combined with accelerator mass spectrometry (Duchateau et al. 2012). Pterodontic acid and the lignan glycosides (LGEO) lack published human pharmacokinetic data whatsoever. Critically, no pharmacokinetic data exist for any compound in this review relating to concentrations achievable in bronchoalveolar lavage fluid (BALF) or lung tissue, the primary site of IAV replication. All preclinical anti‐IAV IC_50_/EC_50_ values were generated in cell culture models that do not replicate the respiratory epithelial absorptive barrier, mucociliary clearance, or pulmonary cytochrome P450 (CYP) enzyme expression encountered in vivo. This constitutes a fundamental and unresolved translational barrier.

Several compounds inhibit CYP enzymes that metabolize co‐prescribed drugs, including oseltamivir, antipyretics such as paracetamol, corticosteroids, and antibiotics used to manage secondary bacterial pneumonia, raising a patient safety concern highly relevant to the anti‐IAV context. EGCG at high supplementation doses inhibits CYP1A2 and CYP3A4 in human liver microsomes (Satoh et al. 2016). The preclinical studies reviewed in this manuscript do not uniformly assess safety at pharmacologically relevant concentrations. High‐dose green tea extract supplementation (> 800 mg EGCG/day) has been associated with hepatotoxicity in clinical reports (EFSA Panel on Food Additives and Nutrient Sources added to Food (ANS) et al. 2018). Carvacrol, evaluated as a TLR7/RIG‐I modulator in the C57BL/6 mouse model, demonstrates embryotoxicity in rodent studies at high doses and mucosal irritancy at elevated concentrations, complicating inhalation delivery strategies.

Apart from plant compounds, several studies discussed rely on crude plant extracts containing chemically complex and often uncharacterized mixtures of bioactive constituents. This represents an important limitation, as it precludes ultimate attribution of observed antiviral or immunomodulatory effects to specific phytochemicals and limits the ability to establish structure–activity relationships. Furthermore, variability in plant source, geographic origin, harvest timing, extraction methods, and composition can significantly affect reproducibility and hinder standardization, thereby constraining translational potential. However, it is also important to recognize that the use of crude extracts is a common and accepted approach in early‐stage natural product research, particularly in ethnopharmacology and nutraceutical studies, where multi‐component formulations may exert biological effects through synergistic interactions. In this context, extract‐based studies provide valuable preliminary evidence of bioactivity and can serve as a basis for subsequent bioassay‐guided fractionation and compound isolation. Nevertheless, a clearer distinction between findings derived from crude extracts and those supported by studies on defined, purified compounds would strengthen the mechanistic interpretation and facilitate the progression toward reproducible and clinically relevant antiviral agents.

Importantly, clinical translation of plant‐derived anti‐influenza interventions remains limited and has primarily involved complex extracts rather than purified compounds. Standardized black elderberry extract (*Sambucus nigra*) has been evaluated in randomized clinical trials, including a Phase IV study, while *Cistus incanus* extract and multicomponent herbal preparations have also been investigated for respiratory infections and influenza (Zakay‐Rones et al. 2004; Kalus et al. 2009; Somerville et al. 2016; Choi et al. 2020; Macknin et al. 2020). However, the clinical findings for these extracts remain limited or inconsistent, and none of the defined phytochemicals discussed in this review has, to our knowledge, progressed to a dedicated clinical trial or received approval as an anti‐influenza therapeutic. This highlights the substantial gap between preclinical evidence for individual phytochemicals and clinical validation.

Although the plant compounds as well as extracts show promise for clinical application, their translation remains limited by several key challenges, including inadequately characterized bioavailability within the respiratory tract, unresolved safety concerns related to potential immunosuppressive effects, and the lack of standardized pharmaceutical‐grade formulations. Addressing these limitations through focused pharmacokinetic studies, formulation optimization such as nano‐delivery systems, and comprehensive toxicological evaluation will be essential to determine whether these candidates can progress from experimental findings to practical clinical use.

## 8 Conclusion and Future Recommendations

The body of evidence reviewed in this manuscript suggests that phytochemicals interact with IAV‐activated innate immune signaling through multiple discrete pathway targets. These compounds are active modulators of the same innate immune signaling networks during IAV infection, that simultaneously drive antiviral defense and, when dysregulated, contribute to immunopathological damage. Across TLR, RIG‐I, MAPK, and NF‐κB pathways, phytochemicals such as quercetin, EGCG, BBR, carvacrol, apigenin, β‐sitosterol, santin, and miquelianin engage shared signaling components, particularly TAK1, TRAF6, and IRF3, in ways that simultaneously shape antiviral function and inflammatory amplitude.

Yet this review also highlights the substantial distance between preclinical promise and clinical translation. Several fundamental problems recur throughout the literature. The most critical is the failure to distinguish between direct pathway modulation and indirect effects secondary to reduced viral replication, a distinction that determines whether a compound is genuinely immunomodulatory or merely reflecting lower viral load. Compounding this, the majority of studies do not identify molecular binding targets, leaving mechanistic interpretation at the level of pathways rather than precise mechanisms. The concentrations used in cell culture models consistently exceed what is pharmacokinetically achievable in human lung tissue through oral administration, a gap that has never been bridged for any compound reviewed here through BALF measurement. And for crude extracts, the active compounds responsible for the observed effects remain largely unidentified, precluding structure–activity analysis and regulatory‐grade standardization.

The path forward requires a change of experimental culture as much as a change of methodology. Molecular target identification through thermal shift assays and docking validation should become standard practice, not aspirational future work. Pharmacokinetic evaluation in mouse respiratory tissue, not just plasma, is essential to validate in vitro data. Complementarily, human airway organoid models also offer a physiologically relevant intermediate between submerged cell line studies and in vivo lung tissue exposure assessment. And for crude extracts, bioassay‐guided fractionation towards defining active constituents should be the expected next step, not an optional follow‐up. If these standards are adopted, the phytochemicals reviewed here, with their broad immunomodulatory activity, structural diversity, and rich pharmacological precedent, represent a real and underexploited opportunity for host‐directed adjunctive therapy in influenza. The field is ready to move forward; what it needs now is the mechanistic rigor to do so responsibly.

## Author Contributions

**Dibakar Chowdhury:** conceptualization, methodology, data curation, formal analysis, validation, investigation, writing – original draft. **Hillary A. Vanderven:** conceptualization, validation, writing – review and editing, supervision. **Phurpa Wangchuk:** conceptualization, validation, writing – review and editing, supervision. **Subir Sarker:** conceptualization, software, validation, supervision, funding acquisition, project administration, resources, writing – review and editing.

## Funding

The authors have nothing to report.

## Ethics Statement

The authors have nothing to report.

## Conflicts of Interest

None declared.

## Supporting information


**Supplementary Table S1:** List of plant‐derived compounds acting on influenza induced immune signalling pathway.
**Supplementary Table S2:** List of plant extracts acting on influenza induced immune signalling pathway.

## Data Availability

No data was used for the research described in the article.
