# Supplementary material for: Dual Antiviral and Immunomodulatory Effects of Phytochemicals in Influenza A Virus Infection: Targeting Key Host Signaling Pathways
Source: Microbiologyopen. 2026 Sep 28;15(5):e70424. doi: 10.1002/mbo3.70424 (PMC13620208; doi:10.1002/mbo3.70424)
Supplement: Supplementary file 1 [file MBO3-15-e70424-s001.docx]

**Dual antiviral and immunomodulatory effects of phytochemicals in influenza A virus infection: Targeting key host signalling pathways**

**Dibakar Chowdhury^1,2^, Hillary A Vanderven^1,2,3^, Phurpa Wangchuk^4,5^, Subir Sarker^1,2,6*^**

^1^Biomedical Sciences & Molecular Biology, College of Medicine and Dentistry, James Cook University, Townsville, QLD 4811, Australia.

^2^Australian Institute of Tropical Health and Medicine, James Cook University, James Cook University, Townsville, QLD 4811, Australia.

^3^Department of Microbiology and Immunology, Peter Doherty Institute for Infection and Immunity, The University of Melbourne, Parkville, VIC 3010, Australia.

^4^College of Science and Engineering, James Cook University, Nguma Bada campus, McGregor Rd, Smithfield, Cairns, QLD 4878, Australia.

^5^Australian Institute of Tropical Health and Medicine, James Cook University, Nguma Bada campus, McGregor Rd, Smithfield, Cairns, QLD 4878, Australia

^6^Department of Microbiology, Anatomy, Physiology and Pharmacology, School of Agriculture, Biomedicine and Environment, La Trobe University, Melbourne, Victoria 3086, Australia.

***Author to whom correspondence should be addressed: subir.sarker@jcu.edu.au**

**Table S1. List of plant-derived compounds acting on influenza induced immune signalling pathway**

| Compound/Extract | Classification | Source Plant | Model (Cellular/ Animal) | Influenza strain | Antiviral effect | Immunomodulatory effect | Activity type | Reference |
| --- | --- | --- | --- | --- | --- | --- | --- | --- |
| Berberine | Alkaloid | *Coptis* *chinensi* Franch. | MDCK, A549, LET1, HAE, RAW 264.7 | A/PR/8/34 (H1N1), A/WS/33 (H1N1) | Inhibit nuclear export of VRNP | Inhibit ERK MAPK activation | Dual | (Cecil, Davis et al. 2011, Botwina, Owczarek et al. 2020) |
| Epigallocatechin gallate (EGCG) | Flavanoid | *Camellia* *sinensis* (L.) Kuntze (Black tea) | Bronchial epithelial  cells (BEAS-2B) | A/PR/8/34 (H1N1) | Blocks viral membrane hemifusion/internalisation | β-defensin 3 upregulated through activation of MAPK pathways. | Dual | (Kim, Kim et al. 2013, Koszalka et al. 2023) |
| (−)-Epigallocatechin-3-gallate | Flavanoid | *Camellia* *sinensis* (L.) Kuntze (Black tea) | MDCK, HEK293T, BALB/c mice | A/PR/8/34 (H1N1), A/Hong Kong/8/68 (H3N2)  , A/Brisbane/59/2007 (H1N1), B/Panama/45/1990, A/Swine/Hebei/108/2002 (H9N2) | Antiviral effect was not measured in this study | Reduce the protein levels of TLR4 and NF-κB | Dual | (Xu, Liu et al. 2017) |
| Apigenin | Flavanoid | *Elsholtzia rugulosa* Hemsl | MDCK, THP-1, A549 | A/PR8/34 (H1N1), A/Jinan/15/90 (H3N2)  and B/ Jiangsu/10/2003, | Separately reduced viral proteins/NA activity | Suppress IAV induced RIG-I activation | Dual | (Xu, Miao et al. 2020) |
| Santin | Flavanoid | *Artemisia rupestirs* L. | THP-1 | A/FortMonmouth/1/1947 (H1N1),  A/Wuhan/359/1995 (H3N2) | Dose-dependent suppression of viral M2 protein/RNA | Decreased IAV-induced phosphorylation of ERK, p38, and JNK | Dual | (Zhong, Wang et al. 2019) |
| Veratric acid, vitexin, and trolline | phenolic acids, flavonoids  and alkaloids, | *Trollius* *chinensis* Bunge | RAW264.7 | A/FM/1/47 (H1N1) | Antiviral effect was not measured | These compounds can disrupt IAV induced TLR3/4/7 signalling | Immunomodulatory only | (Shi, Chen et al. 2020) |
| Quercetin | Flavonoid | *Eupatorium* *fortunei* Turcz. | 16HBE cells (respiratory bronchial epithelial cells) | A/E/13 (H1N1) | suppressed AV mRNA synthesis for the M2, NP, PA, PB2, and NS-1 genes | Suppressing overinduced TLR7 signalling & NF-κB p65 activation | Dual | (Chen, Zhen-You et al. 2012, Choi, Lee et al. 2017) |
| Apigetrin | Glycoside | *Ixeris sonchifolia* ([Maxim.](http://www.wikidata.org/entity/Q77640)) [Hance](http://www.wikidata.org/entity/Q2657692) | MDCK, A549, BALB/c | A/PR8/34 (H1N1) | Directly inhibited replication (EC₅₀ = 0.19 μg/mL) and reduced IAV NP protein | Inhibit IAV induced TLR3/4/7, P38/JNK MAPK | Dual | (He, Ren et al. 2023) |
| Cirsimaritin | Flavanoid, | *Artemisia scoparia* Waldst. & Kit. | THP1 And MDCK | A/FM/1/47 (H1N1), A/tianjinjinnan/15/2009 (H1N1), A/JiangXi/312/2006 (H3N2) | Dose-dependently lowered viral titresas well as M2 protein directly | Downregulate JNK/p38 MAPK and NF-κB  to inhibit IAV infection | Dual | (Yan, Wang et al. 2018) |
| 3,4,5-tri-O-caffeoylquinic acid | Hydroxycinnamic acid derivatives | *Ilex pubescens* Hook. & Arn. | H292 & RAW 264.7 | A/PR/8/34 (H1N1) | Viral replication inhibited at 200 µM but the viral target was not identified | Inhibit TLR 3/7 to reduce type I IFN production | Immunomodulatory | (Wang, Tang et al. 2024) |
| Pterodontic acid | Terpenoid | *Laggera pterodonta* (DC.) Benth. | MDCK & A549 | A/China/LZP/2017(H3N2), A/PR/8/34 (H1N1) | Disrupts ERK-dependent vRNP nuclear export | Inhibit ERK phosphorylation & STAT1 activation, downregulatecaspase-3/7 expression | Dual | (Guan, Li et al. 2017, Wang, Zeng et al. 2019) |
| Miquelianin (quercetin 3-O-glucuronide) | Flavonoid | *Bupleurum chinense DC,*  *Nelumbo nucifera Gaertn* | MDCK, BALB/c mice | H1N1-UI182(mouse adapted strain) from A/Changchun/01/2009 (H1N1) | Directly inhibits replication (EC₅₀ = 107.8 μM); reduction of viral NP expression was observed | Inhibit TAK1 dependent p38 and  JNK activation | Dual | (Li, Shen et al. 2025) |
| DMO-CAP (6-demethoxy-4′-O-methylcapillarisin) | Flavonoid | *Artemisia rupestris* L | MDCK, A549, HEK293T-17, RAW 264.7 | A/FM/1/47 (H1N1), A/Wuhan/359/1995 (H3N2), A/LiaoningZhengxin/1109/2010 (H1N1, oseltamivir resistant strain), and A/HunanZhuhui/1222/2010 (H3N2) | Dose-dependent decline in viral titres and viral M2 protein | Activates MAPK and stimulates IFN-regulated gene expression | Dual | (Zhong, Wang et al. 2019) |
| β-sitosterol | Phytosterol | *Annona cherimola* [Mill.](http://www.wikidata.org/entity/Q312678) | A549 & 293T cells, BALB/c mice | A/PR8/34 (H1N1), A/FM/1/47 (H1N1) mouse-adapted virus, A/HK/8/68(H3N2), A/GZ/GIRD07/09(H1N1) | Showed no activity against A/PR8/34 (H1N1), A/HK/8/68(H3N2), A/GZ/GIRD07/09(H1N1) | Suppress p38 & ERK1/2 MAPK phosphorylation, NF-κB signalling | Immunomodulatory only | (Zhou, Bei-xian et al. 2012) |
| (+)-pinoresinol-O-β-D-glucopyranoside | Lignan glycoside | *Eucommia ulmoides* Oliver | A549, MDCK | A/PR/8/34 (H1N1), A/Hongkong/8/68 (H3N2) and A/Hongkong/Y280/97 (H9N2), A/Guangzhou/GIRD07/09 (H1N1), B/Lee/1940 | Demonstrated CPE reduction against only A/PR/8/34 and A/Guangzhou/GIRD07/09; did not antiviral activity against other strains. | Inactivate p38 MAPK & p65 NF-κB signalling | Dual | (Li , Liang et al. 2019) |
| Sesamin | Lignan, | *Sesamum indicum* L. | PBMC | A/California/07/2009 (H1N1)pdm09 | Reduces NA activity directly | Inhibit JNK/p38/ERK1/2 and cytokines | Dual | (Fanhchaksai, Kodchakorn et al. 2016) |
| Carvacrol | Phenol compound | *Mosla chinensis* Maxim | C57BL/6 | A/FortMonmouth/1/47 (H1N1) | Directly reduced lung viral RNA load *in vivo* | Significantly reduce the protein levels of TLR7, RIG-I, MyD88, and NF-κB | Dual | (Zheng, Wu et al. 2021) |
| Dihydromyricetin | Flavanoid | *Ampelopsis* *grossedentata* (Hand.-Mazz.) J.Wen & Z.L.Nie | MDCK, A549, HEK293T | A/Puerto Rico/8/34 (H1N1),  A/FM-1/47/1 (H1N1), and A/Aichi/2/68 (H3N2) | Reduces plaque formation and binds PB2 polymerase subunit directly | Reduced the expression of TLR3 and TRIF to reduce cytokines | Dual | (Tian, Sang et al. 2020) |
| Eugenol | Phenolic compound | *Syzygium aromaticum* L. | MDCK & A549 | A/ShanTou/169/06(H1N1),A/PuertoRico/8/34 (H1N1), A/ShanTou/1233/06 (H1N1), A/ShanTou/602/06 (H3N2), A/ShanTou/364/05 (H3N2), A/Quail/HongKong/G1/97 (H9N2) | Directly reduced replication at low concentrations | Inhibit and IKK/NF-κB, p38 MAPK & ERK | Dual | (Dai, Zhao et al. 2013) |
| Curcumin | Polyphenol | *Curcuma longa* [L.](http://www.wikidata.org/entity/Q1043) | MDCK & Macrophages; Mice model (unspecified) | A/Taiwan/3530/2001 (H1N1), A/PuertoRico/8/34 (H1N1), A/ShanTou/169/06 (ST169, H1N1), A/ShanTou/1233/06 (ST1233, H1N1), A/ShanTou/602/06 (ST602, H3N2), A/ShanTou/364/05 (ST364, H3N2 | Blocked viral adsorption; reduced lung viral titre *in vivo* | inhibit NF-κB activation, reduced IκBα phosphorylation and recuced cytokine production | Dual | (Xu and Liu 2017, Dai, Gu et al. 2018) |
| Andrographolide | Diterpenoid | *Andrographis paniculata* ([Burm.f.](http://www.wikidata.org/entity/Q716978)) [Wall.](http://www.wikidata.org/entity/Q730310) ex [Nees](http://www.wikidata.org/entity/Q76349) | MDCK, A549, THP-1 & Raw 264.7; C57BL/6 mice | A/PuertoRico/8/34 (H1N1) | Reduced viral loads *in vivo* | Modulated NF-κB and reduced inflammatory cytokine expression | Dual | (Ding, Chen et al. 2017) |

**Table S2. List of plant extracts acting on influenza induced immune signalling pathway**

| Compound/Extract | Source Plant | Major phytochemicals | Model (Cellular/ Animal) | Influenza strain | Action on host cell | Reference |
| --- | --- | --- | --- | --- | --- | --- |
| Water extract | *Eupatorium fortunei* Turcz | Psoralen, angelicin (coumarins); rutin, quercitrin, isoquercitrin, quercetin (flavonoids); chlorogenic acid, caffeic acid (phenolics) | RAW264.7 cells  (Murine Macrophage) | A/PR/8/34 (H1N1) | Upregulates the  phosphorylation of IRF-3, STAT1, and TBK1in macrophage | (Choi, Lee et al. 2017) |
| Oligonol | *Litchi chinensis* [Sonn.](http://www.wikidata.org/entity/Q507034) | Epicatechin, epicatechin gallate, epigallocatechin gallate (EGCG), procyanidins A1/A2/B1/B2 (proanthocyanidin oligomers) | MDCK | A/Hong Kong/2/68  (H3N2) | Blocking nuclear export through inhibiting ERK activation | (Gangehei, Ali et al. 2010) |
| Sesquiterpene-Rich Extract (SECA) | *Carpesium abrotanoides* L. | Carabrol, carabrone, carpesiolin, ivaxillin, telekin (sesquiterpene lactones) | MDCK | A/PR/8/34 (H1N1) Mouse adapted strain | Reduced TLR4, MyD88 and NF-κB expression | (Li, Yang et al. 2022) |
| Root extract | *Isatis indigotica* Fortune | Polysaccharides | 16HBE & MDCK cells | A/PR/8/34 (H1N1), A/California/07/2009 (H1N1)pdm09, A/Guangzhou/GIRD07/09 (H1N1), A/Aichi/2/68 (H3N2) | Inhibit TLR3 to prevent cytokine storm | (Li, Li et al. 2017) |
| Crude phenylethanoid glycosides (PhG) | *ligustrum purpurascens* Y.C. Yang | Acteoside (verbascoside), isoacteoside, ligupurpuroside A, osmanthuside B (phenylethanoid glycosides) | MDCK, C57BL/6J mice | A/FM/1/47 (H1N1) | Inhibit IAV by endogenous interferon-gamma (IFN-γ) production | (Hu, Shao et al. 2016, Song, Li et al. 2012) |
| Sesquiterpene fraction | *Laggera pterodonta* (DC.) Benth. | Pterodontic acid, pterodondiol (sesquiterpenes) | MDCK & A549 | A/PR/8/34 (H1N1), A/Aichi/2/68 (H3N2) | Inhibit p38 MAPK/ p65 NF-κB | (Wang, Zhou et al. 2017) |
| Total Flavonoids (MSTF) | *Mosla scabra* (Thunb.) C.Y.Wu & H.W.Li | Luteolin, apigenin, kaempferol, luteoloside, mosloflavone I/II (flavonoids) | ICR mice | A/PR/8/34 (H1N1) | Suppressing MAPK, NF-κB and proinflammatory cytokines | (Yu, Yu et al. 2016, Cai and Zhang 2022) |
| Total flavones (TFA) | *Abelmoschus manihot (L.)* Medik. | Hyperoside, isoquercetin, quercetin-3′-O-glucoside, myricetin, quercetin, rutin (flavone glycosides) | MDCK & Mouse alveolar  macrophage | A/PR/8/34 (H1N1) | Suppress MAPK and cytokines | (Gao, Liang et al. 2022) |
| AM Injection (AMI) | *Astragalus membranaceus* Moench | Astragalus polysaccharides (APS), astragaloside IV, calycosin-7-O-glucoside | Raw264.7& MDCK | A/FM/1/47 (H1N1) | Modulating the TLR3–TBK1–IRF3 signalling, reduce oxidative stress | (Liang, Zhang et al. 2019) |
| Total flavonoids | *Mosla chinensis* Maxim | Luteolin, apigenin, | ICR | A/PR/8/34 (H1N1) | Suppress TLR3, TLR7, NF-κB p65 & oxidative damage | (Zhang, Wu et al. 2018)) |
